# Epileptiform activity in the mouse visual cortex interferes with cortical processing in connected areas

**DOI:** 10.1038/srep40054

**Published:** 2017-01-10

**Authors:** L. Petrucco, E. Pracucci, M. Brondi, G. M. Ratto, S. Landi

**Affiliations:** 1 NEST, Istituto Nanoscienze CNR and Scuola Normale Superiore Pisa, Pisa, Italy

## Abstract

Epileptiform activity is associated with impairment of brain function even in absence of seizures, as demonstrated by failures in various testing paradigm in presence of hypersynchronous interictal spikes (ISs). Clinical evidence suggests that cognitive deficits might be directly caused by the anomalous activity rather than by its underlying etiology. Indeed, we seek to understand whether ISs interfere with neuronal processing in connected areas not directly participating in the hypersynchronous activity in an acute model of epilepsy. Here we cause focal ISs in the visual cortex of anesthetized mice and we determine that, even if ISs do not invade the opposite hemisphere, the local field potential is subtly disrupted with a modulation of firing probability imposed by the contralateral IS activity. Finally, we find that visual processing is altered depending on the temporal relationship between ISs and stimulus presentation. We conclude that focal ISs interact with normal cortical dynamics far from the epileptic focus, disrupting endogenous oscillatory rhythms and affecting information processing.

Electrographic seizures (*i.e.* ictal events) are regarded as the clinical sign of an active epileptic condition, while interictal activity is defined as the multiform cluster of electrophysiological signatures occurring between consecutive seizures and manifestly distinguishable from normal brain activity^1^. In the broad definition of interictal activity, hypersynchronous interictal spikes (ISs) are quite common features of epileptiform activity with large ensemble of neurons periodically firing hypersynchronous potentials^2^,^3^. Although often critical, seizures may appear only sporadically with no clear leftovers, while interictal activity may lurk in an apparently normal brain even in the absence of any record of past ictal events and without overt neurological symptoms^4^. Since during an IS, neuronal firing is hijacked by the pathological rhythm, local computation is obviously altered and ISs can cause cognitive deficits even in absence of seizures^5^,^6^,^7^,^8^,^9^,^10^. Indeed, studies based on EEG observations concluded that ISs can lead to transitory cognitive effects in humans^11^,^12^,^13^,^14^,^15^. For example, driving ability is impaired by ISs since they extend reaction time in simulated driving tests^16^, and serious effects of ISs are evident in children where ISs are correlated to attentional deficits and poor scholastic performances^17^.

Most studies addressed the impact of ISs on neuronal computation occurring in the epileptogenic region. However, we should also expect that focal ISs can affect brain functions in a more subtle and extensive way, by propagation through long-range connections to areas not directly engaged in epileptiform activity^18^,^19^. In fact, coupled EEG and fMRI recordings in human subjects have shown that changes of the BOLD signal are present in remote structures far from the epileptic generator^20^,^21^.

In light of these considerations, here we studied how focal ISs interfere with neuronal processing of connected areas. Focal IS activity was triggered in the visual cortex of the anesthetized mouse by strictly localized superfusion of the GABA_A_ antagonist bicuculline^22^,^23^,^24^,^25^,^26^. Local delivery of this drug induced patterns of interictal activity resembling the one appearing in lesional human epilepsy^27^ and in idiopathic benign partial epilepsies of childhood^28^,^29^. Indeed, bicuculline has been extensively used in studies addressing the mechanisms of IS generation^30^,^31^,^32^. By recording *in vivo* local field potentials (LFP) and single-cell firing by loose patch-clamp we determined that even if ISs did not invade the opposite hemisphere, the EEG was subtly disrupted, with a decrease in length and an increase in frequency of up states. By phase-locked averaging of the LFP we found that every IS caused a peculiar change of the firing probability of the contralateral hemisphere during slow-wave activity. Initially the ISs facilitated firing in this area but, after about 100 ms, the cortex was completely silenced for almost 300 ms leading to a precocious termination of the up state. Finally, we determined that visual evoked responses were affected by ISs depending on the temporal relationship between stimulus presentation and contralateral spike burst.

Together, our data prove that ISs interact with other cortical dynamics far from the epileptic focus, disrupting endogenous oscillatory rhythms and affecting brain information processing. This evidence sustains the notion of an IS-induced cognitive impairment in local and distant areas, an idea that has major clinical implications in the ongoing discussion about the pharmacological treatment of subclinical EEG anomalies^15^,^33^,^34^, especially in children, where cognitive impairment induced by ISs is more severe^35^,^36^. Here we studied the effects of acute IS induced in a naïve brain on cortical processing and we determined that brain computation is strongly affected even in absence of the circuitry rearrangements proper of chronic epilepsy.

## Results

### Slow-wave oscillations are synchronized between hemispheres

**Figure 1:**
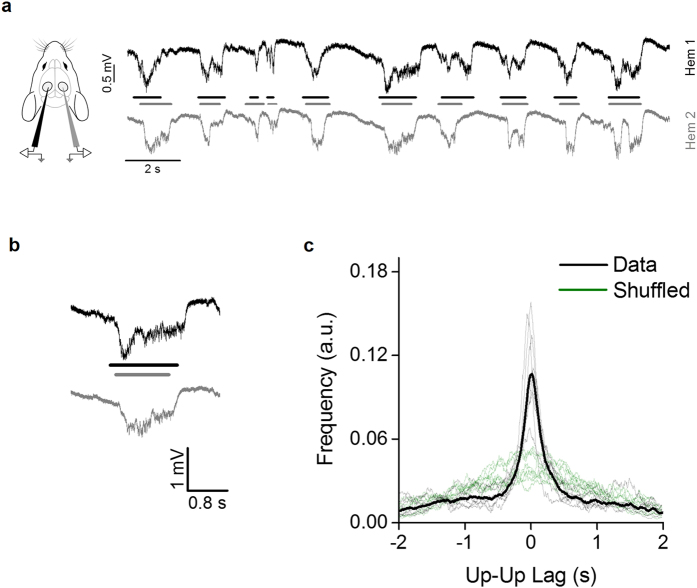
Slow-wave activity is synchronized between the two hemispheres. (**a**) Examples of baseline activity in the urethane anesthetized mouse in the two hemispheres (*black* line: Hem 1, *gray* line: Hem 2). (**b**) Magnification of a single up state recorded simultaneously in both hemispheres. (**c**) The distribution of lag times between the middle point of up states in the two hemispheres (*dashed lines*: single animals; *continuous line*: average), compared to the distributions obtained after randomly shuffling up states sequence in Hem 2 (*green traces*). The shuffling returns a far more dispersed distribution indicating the correlation between the two hemispheres.

First, we recorded baseline activity in both hemispheres in the primary visual cortex (V1) of C57BL/6 J mice under urethane anesthesia. In accordance with previous reports we observed slow-wave oscillations typical of non-REM sleep and resting wakefulness, with alternating up and down states^37^,^38^. Up states were characterized by a negative deflection of about 0.8 mV in the recorded extracellular potential (Fig. 1a,b). The mean duration of up states ranged between 0.45 and 0.75 s for all animals, and the mean frequency between 0.55 and 0.95 Hz. We observed that up and down states pattern appeared to be synchronized between the two hemispheres, as previously reported^39^,^40^. We measured the temporal delay between the middle point of each up state in hemisphere 1 (Hem 1; Fig. 1a,b, black line) with the nearest up state middle point in hemisphere 2 (Hem 2; Fig. 1a,b, gray line); then we computed the distribution of the time lag between the nearest neighbor (NN). To test whether this distribution supports the up states synchronization between hemispheres, we generated a shuffled sequence of up and down states in one hemisphere and we computed the NN distribution with this new sequence. The comparison between original and shuffled NN distributions demonstrates a significant difference (KS test, p < 0.001 for each animal), proving that the oscillations are coupled (Fig. 1c). Since the observed distributions are symmetrical, we concluded that there was no dominant hemisphere in the generation of slow-wave oscillations.

### ISs fragment the up states in the opposite hemisphere

**Figure 2:**
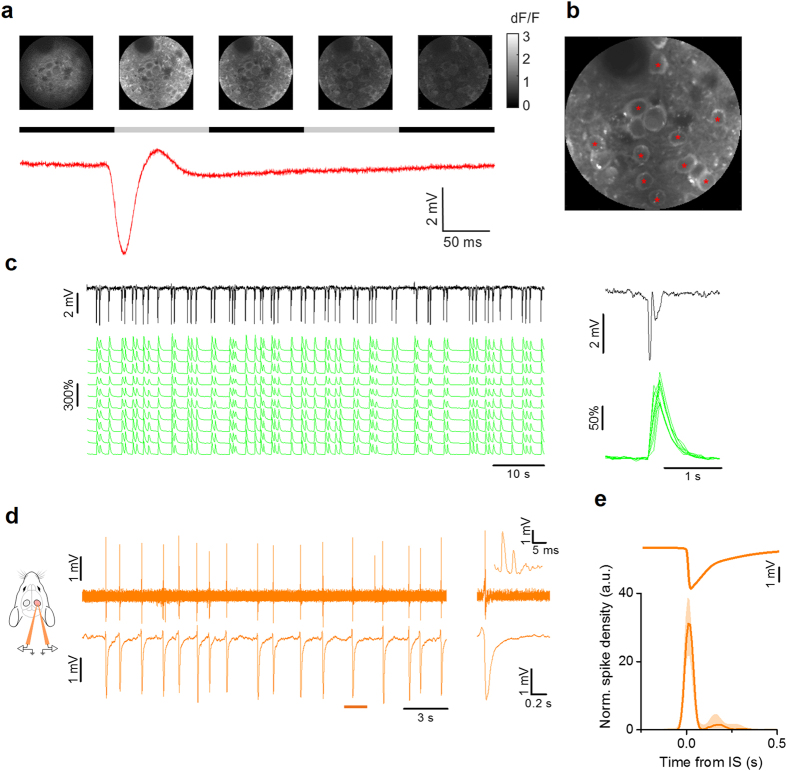
Single cell dynamics during IS activity in V1. (**a**) Sample images obtained during *in vivo* two-photon imaging in the visual cortex of a CaMKII-GCaMP6f mouse. The time lapse sequence is over imposed to the simultaneous recording of the extracellular potential and shows that ISs are accompanied by a calcium transient that occurs simultaneously in all neurons in the field. The horizontal black and gray bars show the sampling period for each frame. (**b**) Sample ΔF/F traces measured for neurons (*red asterisks*) in the field. (**c**) The LFP is aligned to the calcium responses. The inset shows the stereotyped Ca^2+^ transients recorded during a single IS. (**d**) Sample loose-patch recording from a L2/3 neuron during interictal activity (*upper trace*) and the simultaneous LFP recording from a nearby extracellular electrode. The inset shows the neuronal firing during a single IS event, with an enlargement around the IS onset. (**e**) Spike density mediated on all recorded neurons aligned to the ISs after normalization with mean firing frequency (median +/− quartiles, n = 9 cells from 6 mice). The vast majority of action potentials are fired within a 100 ms wide interval centered on the IS (*horizontal bar*).

Interictal activity was elicited focally in the visual cortex of Hem 2 by localized superfusion with bicuculline (BMI) 100 μM. As already described^9^, the focal treatment with bicuculline was followed within minutes by the appearance of ISs, characterized by stereotypical sharp deflections (2–6 mV) of the LFP recording that remained localized in the treated cortical area (Fig. 2c, *black line*). ISs implicated the transient and simultaneous recruitment of most neurons in the affected focus, as demonstrated by *in vivo* two-photon imaging (Fig. 2a–c). *In vivo* loose-patch recordings showed that neuronal firing occurred in a window of about 50 ms centered on the onset of the LFP transient. Each neuron produced only a small number of action potentials for each IS (median 2.6 spikes/IS (1° quart: 1.9, 3° quart: 4.0), n = 9 neurons from 6 mice; Fig. 2d,e). The interval between two consecutive ISs had a wide non normal distribution (Lilliefors normality test, p < 0.001), with a median interval of 2.6 s (0.38 Hz; n = 8 mice).

**Figure 3:**
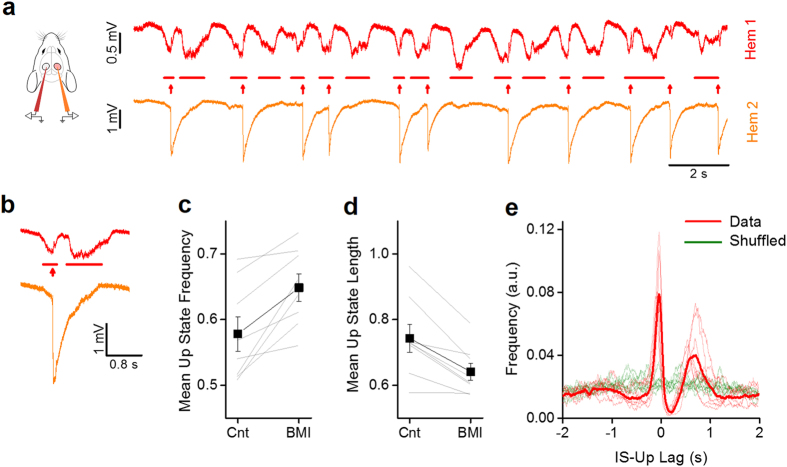
Focal ISs alter the slow oscillations in the opposite hemisphere. (**a**) Traces showing activity in Hem 1 when in Hem 2 focal ISs are induced by localized application of BMI 100 μM. (**b**) Magnification of a single up state and a corresponding IS recorded simultaneously in the two hemispheres. (**c**) The mean frequency of up states is increased after BMI in Hem 2. The thin lines indicates the change in mean frequency in the same mouse before and during the BMI treatment (Cnt, 0.57 ± 0.03 vs. BMI, 0.64 ± 0.02, paired t-test, n = 8, p = 0.004). (**d**) The duration of up states is shortened by ISs in Hem2 (Cnt, 0.74 ± 0.04 vs. BMI, 0.64 ± 0.03, paired t-test, n = 8, P = 0.003). (**e**) Distributions of lag times between the middle point of up states in Hem 1 and the onsets of IS events (*dashed lines*: single animals; *continuous line*: average). The double peak in the distribution shows that the ISs follow the up states with a small jitter evidenced by the small dispersion of the first left peak. The broader peak on the right is due to the reappearance of the up state that follows the IS with a more variable duration, as evidenced by the broader distribution. Notice that ISs are never over imposed to up states.

First of all, as evidenced in Fig. 3a,b, there was a transient interruption of the ongoing up state by the emerging IS. The presence of ISs in Hem 2 was associated with a change of the statistics of the slow-wave oscillations in Hem 1; in particular, as shown in Fig. 3c,d, the frequency of up states increased from 0.70 ± 0.06 Hz to 0.81 ± 0.06 Hz (paired t-test, n = 8, p = 0.007), while their duration decreased from 0.74 ± 0.04 s to 0.64 ± 0.03 s (paired t-test, n = 8, p = 0.003).

These data suggested a bidirectional interaction between up states and ISs: the onset of an up state caused an IS, which in turn, fragmented the up state in the contralateral cortex. To verify this hypothesis, we computed the distribution of the time lag between each IS and the middle point of the nearest up state in Hem 1 (Fig. 3e). The lag distribution showed a peculiar double peak and a strong decrement in correspondence of the zero-lag point. This suggests that the majority of ISs occurred immediately after the onset of an up state and that the ISs in Hem 2 caused the interruption of the up state in Hem 1. The random temporal shuffling of the up state sequence led to completely different distributions of lags (*green lines* in Fig. 3e), demonstrating the correlation between the two processes.

**Figure 4:**
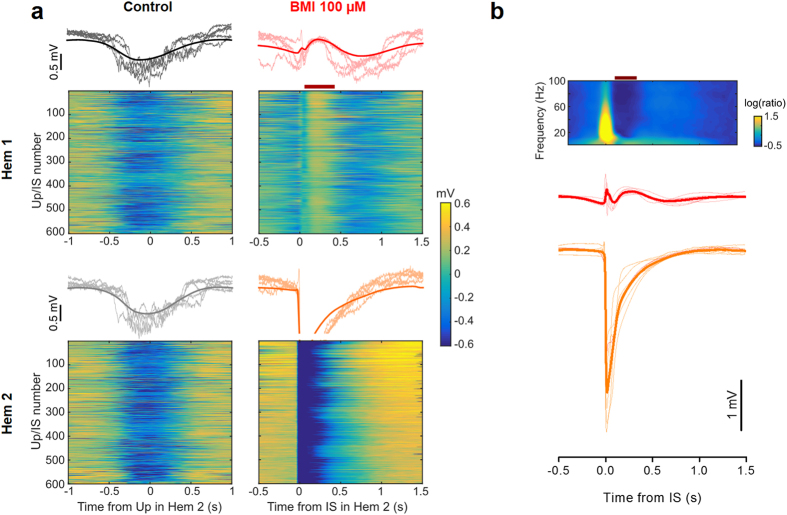
Contralateral ISs disrupt slow-wave oscillations. (**a**, *top*) Raster plot of LFP segments recorded in the two hemispheres, aligned to the middle point of the up state detected in Hem 2. The thick line represent the mean of the representative traces. Notice the synchronization between up states in the two hemispheres. (**a**, *bottom panel*). Raster plot of LFP segments in the two hemispheres, aligned to the IS onset, and their average. The raster plot clearly shows the window of reduced firing that appears immediately after each IS (*horizontal red bar*), which is followed by a new up state. (**b**) Mean traces of up state waveforms phase-locked with the beginning of ISs in Hem 2. Data recorded in 8 mice (*dashed lines*) and their average (*continuous line*). The silencing period is always present. Above is shown the average spectrogram of the LFP segments in Hem 1, phase-locked on the IS. The window of reduced activity is clearly associated to a strong reduction in the spectral power (*horizontal red bar*).

To analyze further this relationship, we divided the LFP recorded in Hem 1 in short segments centered on the onset of each IS. The raster plots in Fig. 4a represent the cropped LFP recorded from the two hemispheres of one representative mouse. The interruption of the up states in Hem 1 following the IS in Hem 2 was a consistent phenomenon (see *red bar* in Fig. 4a, lower left), as it occurred in every fragment. The average field shown above the raster plot clearly reports the temporal dynamic of the interplay between up states and ISs: each IS was preceded by an up state onset, and in turn caused a brief, transient interruption of the up state. In average, the lag between the up state and the following IS was about 200 ms and the up state interruption caused by the IS lasted for about 300 ms. A characteristic small deflection of the LFP was observed in Hem 1 in correspondence to the IS. Figure 4b reports the mean of the IS-locked LFPs of every recorded animal (*thin lines*) and the population mean.

We analyzed the LFP segments in the frequency domain by computing the average spectrogram of the LFP segments (Fig. 4b, *top*). The up state interruption was clearly visible as a reduction in all frequencies up to 100 Hz (*red bar*), and in correspondence of the interictal peak there was a sharp increase in the power of all frequencies. We supposed that at this time Hem 1 was receiving a transient excitatory stimulation from the contralateral cortex, with an increased probability of neuronal firing.

### ISs alter the neuronal firing probability in the contralateral cortex

**Figure 5:**
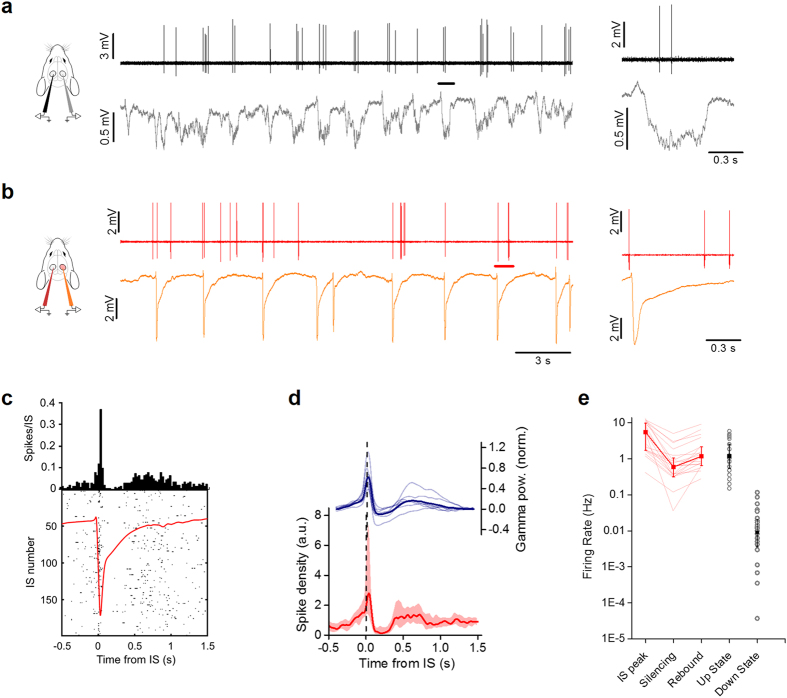
ISs modulate the firing probability in the contralateral hemisphere. (**a**) Loose-patch recording from one neuron in Hem 1 (*upper black line*) and contralateral LFP in Hem 2 (*lower gray line*) displaying sparse firing during slow oscillations. Insert shows a magnification of firing activity during a single up state (*black bar*). (**b**) The same neuron has been recorded (*upper red line*) during focal IS activity (LFP, *orange trace*) caused by focal superfusion with BMI in Hem 2. The inset shows a magnification of a single IS. (**c**) Peri-IS raster plot showing spike pattern during 200 different ISs. The upper diagram is the spike distribution computed from the raster plot. The distribution has a sharp peak centered on the peak of the contralateral IS (red trace) that is followed by a period of reduced firing. (**d**) The red trace is the median distribution of firing for all the recorded cells in Hem 1 phase-locked to the ISs occurring in Hem 2 (median +/− quartiles, n = 20 from 6 animals). The blue trace represent the power in the gamma band (40–100 Hz) of the LFP in Hem 1 (n = 7 mice; *thick line*: mean, *thin lines*: single experiments). (**e**) Firing rates in different stages during contralateral ISs. The firing rate during rebound is extremely close to the firing rate during the up state (rebound: 1.18 spikes/s, n = 20 neurons from 6 mice; up state: 1.19 spikes/s, n = 28 neurons from 14 mice; p = 0.81, KS test).

In order to verify that contralateral ISs changed neuronal firing probability, we performed loose-patch recordings of neurons in Hem 1 (Fig. 5a,b). As previously described^41^, before superfusion of bicuculline each neuron fired up to a few action potentials only during each up state (Fig. 5a). When bicuculline was added in Hem 2, we observed a biphasic effect on the firing probability in Hem 1 (Fig. 5c,d). Interestingly, the temporal pattern of the firing density is perfectly mirrored by the temporal evolution of the gamma band power (Fig. 5d). In correspondence with the IS peak, the firing probability was briefly boosted and this facilitation was immediately followed by a window of decreased firing of about 0.3 s, which overlapped perfectly with the silencing observed in the field recordings. Between 0.3 and 0.8 s after the IS, the firing rate incremented again, following the reappearance of the up state observable in the field potential (Fig. 5e). These data showed that contralateral ISs modified the temporal pattern of spontaneous discharges in the control hemisphere. This happened without a large increase in the overall number of spikes (Cnt: median 1.72 spikes/s, n = 28 neurons from 12 mice; BMI: 2.00 spikes/s, n = 20 neurons from 6 mice; KS-test: p = 0.1). In conclusion, ISs strongly modulated the density of firing probability in a brief time window, thus suggesting a transient alteration of cortical computation and, presumably, of the processing of visual stimuli.

### Contralateral interictal spikes interfere with visual processing

**Figure 6:**
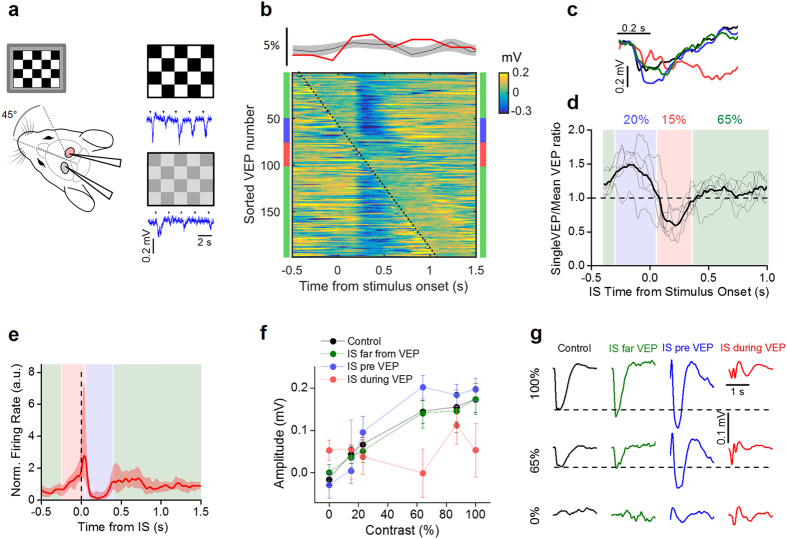
ISs interfere with normal visual processing. (**a**) Schematic of the experimental setup and exemplificative traces at two different contrasts. (**b**) The upper panel shows the probability distribution of IS onset in respect of the stimulus (*red trace*: lower panel experiment; *gray area*: mean +/− SD for n = 8 animals). The lower panel shows the raster plot obtained by sorting VEP traces according to the delay between stimulus and IS. The dotted black line represents the appearance of IS; time 0 indicates the stimulus presentation. (**c**) Black trace reports a single response to a stimulus presentation in the control recording (before of BMI superfusion), the blue and red traces report the responses when IS immediately preceded (−0.35 to 0.05 s, *blue*) or followed (0.05 to 0.35 s, *red*) stimulus presentation; in green there are the mean traces obtained when the stimulus and the closest IS were farther away than 0.35 s. These windows are indicated by the colored bars adjacent to the raster plot in **b**. (**d**) VEP amplitudes depend on the lag between stimulus and contralateral ISs (N = 6 mice; average in thick line, each mouse in thin lines). The colored shaded zones span the time windows used for the averaging in **c**. The percentages report the fraction of stimuli falling in the three conditions averaged on all mice. (**e**) Periods of VEP alteration have been superimposed on the peri-IS spike histogram of Fig. 5d with the same color code in **d**, to show the relationship between the effect of ISs on neuronal firing and VEP alteration. (**f**) Contrast sensitivity for the three groups of responses compared to control (n = 8 mice). Curves from the VEPs falling after the ISs (IS pre VEP) and for the VEPs interrupted by the ISs (IS during VEP) showed a significant difference, while curve from the VEPs far from the ISs was no significantly different from control (t-test, p-value > 0.05). (**g**) Mean VEP waveforms recorded in the same mouse at different contrasts for the different conditions reported in **f**.

We evaluated the processing of visual stimuli by recording the visual evoked potentials (VEPs) in V1, in response to the periodic reversal of a checkerboard (2 s period; Fig. 6a). The probability distribution of the timing of the ISs relative to the checkerboard reversal clearly demonstrated that visual stimuli did not influence IS statistics (upper panel of Fig. 6b). To evaluate the effects of ISs on sensory responses, we sorted the records containing the VEPs according to the distance of the stimulus onset from the nearest IS occurring in Hem 2. A raster plot of the records from one single animal is depicted in the lower panel of Fig. 6b. The checkerboard reverses at 0 s and the VEPs are represented by the vertical blue band that appears with a latency of about 150 ms from the stimulus onset; the dotted line marks the timing of ISs. As exemplified in this plot, ISs had an effect on the VEPs depending on their relative timing: ISs falling shortly before the stimulation caused an increment in the recorded potential compared to the VEPs recorded in baseline conditions, while ISs occurring immediately after the stimulation suppressed the evoked potential (see Fig. 6b,c). We quantified this effect by calculating the VEP amplitude in response to a high contrast checkerboard as a function of the temporal distance between the IS and the stimulus presentation (Fig. 6d). In every experiment we observed an enhancement of the response (*blue band*) followed by a strong suppression (*red band*). When the distance between reversal and the closest IS was larger than about 350 ms, the response was similar to the response measured before BMI superfusion (*green bands*). These temporal windows are reported in Fig. 6e together with the effect of ISs on the firing rate (replotted from Fig. 5d). The firing increase caused by ISs, masked the external stimulus, interrupting the ongoing VEP and causing a brief window of functional blindness. On the other side, the following silencing appeared to increase the sensitivity of the cortex to the visual stimulus. A similar behavior has been observed for sensory stimulations elicited during the down state, especially for high-intensity stimuli^42^,^43^, strengthening the idea that during this time window the contralateral cortex switched to a down state.

To assess the ability of V1 of extrapolating significant features of visual stimuli in presence of contralateral IS activity, we measured the contrast sensitivity by recording the evoked potentials in response to checkerboards of variable contrast^44^. We divided the recordings in three groups, according to the interval between the stimulus and the closest IS. The first group was represented by records in which the stimulus was farther away from any IS than 350 ms (*green zone* in Fig. 6d,e). The second group (*blue zone*) included records in which the IS immediately preceded (−0.35 to 0.05 s) the stimulus. Finally, the third group (*red zone*) included records in which the IS followed (0.05 to 0.35 s) stimulus presentation.

The contrast sensitivity curves in the three conditions are shown in Fig. 6f and were calculated by measuring the integral of the VEPs in a window of 0.4 s from their onset (0.1 s after stimulus presentation). As it can be seen in Fig. 6g, occurrence of an IS before or after the presentation of a visual stimulus significantly altered the detection of different contrasts. In our experimental conditions, visual processing was disrupted intermittently for about 35% of the time (Fig. 6d). These data demonstrated that each focal IS transiently influenced signal processing in wide cortical territories following a complex biphasic modality dependent on the relative timing between IS and stimulus in homotypic regions of different hemispheres.

## Discussion

Epilepsy is a multiform pathology with variable etiology and clinical development^1^. In consequence of that, it is difficult to reproduce it in animal models, because there are several transgenic or pharmacological models for most part of diagnosed forms of epilepsy, while many types of diseases remains still to be investigated. Aim of our study is to investigate how an abnormal acute activity induced in a previously normal brain affects information processing in cortical areas far from the epileptiform focus in the anesthetized animal.

It is known that epilepsy produces cognitive impairment at different levels according the magnitude and frequency of seizures and the areas involved; these deficits are usually temporary, but their severity correlates with developmental onset time^45^, being more severe in children. Interestingly, it has been seen that chronic interictal activity in children can be comparable to epilepsy with seizures in terms of cognitive deficits, even if its effects can be less pronounced in number of areas involved and in extension^6^.

Here, we describe two different mechanisms that can affect cortical function in areas connected to foci of epileptiform activity. Firstly, we observe that ISs interact with slow-wave activity, fragmenting up states and potentially interfering with their role on the homeostasis of cortical circuitry. Secondly, ISs interfere with cortical computation by introducing an external non-physiological modulation of neuronal excitability in correspondence of every contralateral IS.

Cortical slow-wave oscillations are critical for memory consolidation and brain homeostasis^46^,^47^ and alterations of this rhythm potentially lead to long-term cognitive dysfunctions. Our data suggest that the same cellular mechanism causing the up state onset in Hem 1, triggers an IS in Hem 2 after a brief and variable lag. Thus, slow-wave activity promotes ISs, as also demonstrated in human patients^48^. In turn, the IS closes prematurely the up state, forcing the cortex in a brief down state, thus fragmenting the slow-wave cycle. Recently, a similar alteration has been found in a chronic rat model of hippocampal interictal epileptiform discharges, suggesting the generality of this mechanism^49^. There are also clinical evidences pointing toward the importance of a disruption of the sleep slow oscillation in the emergence of pathological conditions; for example, in children affected by Landau-Kleffner syndrome^50^,^51^,^52^, a form of partial epilepsy with continuous spikes and waves during sleep (>85% of time), there is regression of cognitive function, long-standing developmental delay and loss of acquired language skills that are irreversible after two years from appearance of symptoms. In this disease, the putative role of horizontal connectivity is supported by the ameliorative effects of intracortical resection^53^,^54^.

The second question we address in our study regards the impact of epileptiform activity on interconnected regions of the brain not directly interested by the focus. Although early reports have already shown that neurons in the contralateral cortex may display altered activity after the induction of an interictal focus^18^,^19^, the relationship of this alteration with up and down states as well as its effects on visual processing are still unknown. Our experiments evidence a biphasic effect of ISs on contralateral activity: after a brief time period of increased firing in correspondence with the IS a window of strongly reduced firing appear. The effects on the responses to visual stimuli mirror this dual effect of ISs on spontaneous firing. Indeed, the subdivision of VEPs according to their temporal connection with ISs demonstrate that ISs can enhance or suppress visual responses depending on their temporal relation with the stimulus. Given the average frequency of ISs in our model, the visual cortex operates in an altered state for about 35% of time. This would be hardly compatible with a proper control of visually driven behavior. Indeed, several papers have shown that electrophysiological estimations of visual properties correlate with behavioral analysis^55^,^56^,^57^,^58^,^59^. However, further studies could be done to better investigate perception in these epileptiform mice in future.

Furthermore, we can speculate that if interictal spike activity is generated in one associative area, its propagation to the contralateral may affect both local associative functions and possibly, related sensory perception. Conversely, propagation of interictal events into primary sensory cortices might be responsible of sporadic neglect or alteration of bottom-up sensory coding. These phenomena might be triggered by focal epileptiform activity localized millimeters away from the control region, leading to a revision of the concept of “focal activity” and possibly promoting a more brain-wide approach to epilepsy treatment.

## Methods

### Mouse preparation

Adult (age > postnatal day 60) C57BL/6 J mice were used (n = 26). Animals were reared in a 12 h light/dark cycle, with food and water available *ad libitum*. All experimental procedures conformed to the European Communities Council Directive n° 86/609/EEC and were approved by the Italian Ministry of Health.

Recordings were performed as described previously^60^,^61^. Mice were anesthetized by intraperitoneal injection of urethane (0.8 ml/hg in 0.9% NaCl; Sigma). Additional doses (10% of initial dose) were intraperitoneally administered to maintain the anesthetic level when necessary. The head was fixed in a stereotaxic frame. Body temperature during the experiments was constantly monitored with a rectal probe and maintained at 37 °C with a heating blanket. The depth of anesthesia was evaluated by monitoring pinch withdrawal reflex and other physical signs (respiratory and heart rate). A portion of the skull overlying the visual cortex (0.0 mm anteroposterior and 2.7 mm lateral to the lambda suture) was drilled on both the hemispheric sides and the *dura mater* was left intact. A double chamber was created with a thin layer of a synthetic resin (Paladur, Heraeus Kulzer GmbH & Co.) around the edges of the craniotomy. Cortex was maintained constantly wet with artificial cerebrospinal fluid (ACSF) solution: NaCl 132.8 mM, KCl 3.1 mM, CaCl_2_, 2 mM, MgCl_2_ 1 mM, K_2_HPO_4_ 1 mM, HEPES 10 mM, NaHCO_3_ 4 mM, glucose 5 mM, ascorbic acid 1 mM, myo-inositol 0.5 mM, pyruvic acid 2 mM, pH = 7.4. In order to induce interictal spikes, the solution inside one chamber was replaced by ~40–60 μL of GABA_A_ receptor agonist bicuculline methiodide (BMI, 100 uM in ACSF solution; Sigma). The drug superfusion was restricted to a single hemisphere and verified by means of paired local field potentials recordings. BMI was occasionally added in order to keep constant the interictal activity pattern. Animals deeply anesthetized under urethane were sacrificed by cervical dislocation without regaining consciousness at the end of the experiment.

### Local field potential and loose-patch recordings

To record local field potentials LFPs in the two hemispheres, two glass micropipettes (impedance ~2 MΩ, filled with ACSF solution) were positioned into the visual cortex at a depth of 250–300 μm (II/III layer) with a motorized micromanipulator (MPI electronic). A common reference Ag-AgCl electrode was placed on the cortical surface.

For the loose-patch recordings^62^ glass micropipettes (4–8 MΩ resistance, filled with ACSF; Sigma) and an axopatch-1D amplifier were used. The pipette was inserted through the pia by applying about 300 mbar of positive pressure until II/III layer was reached^63^. Cells were searched in voltage-clamp mode with the positive pressure lowered to 30 mbar while monitoring the tip resistance with a square-wave current pulse (test stimulus 20 mV). On approaching a cell, pressure was relieved and light suction was applied. Voltage responses were recorded in current clamp mode (I = 0) with a 10-fold gain.

Electrophysiological signals were amplified 1000-fold (EXT-02F, NPI), band pass filtered (0.1–1000 Hz), and sampled at 10 kHz with 16 bit precision by a National Instruments (NI-usb6251) AD board controlled by custom made LabView software. Line frequency 50 Hz noise was removed by means of a linear noise eliminator (Humbug, Quest Scientific).

### Two-photon calcium imaging combined with LFP recordings

Imaging was performed on adult mice (P > 90) obtained by crossing a homozygous B6;Cg-Tg(CaMKIIa-cre)T29-1Stl/J female (Jackson lab, stock number #005359) with a B6;129 S-Gt(ROSA)26Sor^tm95.1(CAG-GCaMP6f)Hze^/J homozigous male (Jackson, stock number #024105). In this animal (CaMKII-GCaMP6f) the expression of the genetically encoded Ca^2+^ sensor GCaMP6f was restricted to pyramidal cells of LII/III. The mouse was head-fixed and a craniotomy of 2–3 mm in diameter was drilled over the visual cortex as for electrophysiological experiments. A perforated glass was glued to the craniotomy in order to facilitate the penetration of drugs after the acquisition of the baseline activity of the brain and to record LFPs. Imaging was performed with a two-photon microscope (Ultima IV, Prairie Technology) equipped with a 18 W laser (Chamaleon Ultra 2, Coherent) tuned at 890 nm that delivered about 30 mW at the sample. Images were acquired 200–250 μm below the cortical surface with a water immersion objective (Olympus XLUMPLFLN-W 20X, numerical aperture) at a resolution of 512 * 512 pixels at 10 Hz (spiral scan). The same field was acquired in baseline condition and after administration of BMI 100 μm.

### Visual evoked potentials (VEPs)

VEPs in response to alternating checkerboards modulated at different contrasts were recorded at the same depth of LFPs. All visual stimuli were computer-generated on a display (mean luminance at maximum contrast, 3 cd/m^2^) by a MATLAB custom script that exploits the Psychophysics Toolbox. The luminance of the checkerboard was calibrated by means of a photometer (Konica Minolta). The contrast values were calculated with the Michelson’s formula: (I_max_ − I_min_)/(I_max_ + I_min_)^64^. Transient VEPs were recorded in response to the reversal (0.5 Hz) of the checkerboard (spatial frequency 0.04 c/deg). The response to a blank stimulus (0% contrast) was also recorded to estimate noise.

### Data analysis

Collected data were visually inspected, and traces presenting drift or other artefacts were excluded (~1% of the data). All the subsequent analysis was performed on MATLAB (The MathWorks Inc.). We designed a custom software for the automatic detection and measurement of up and down states based on the computation of spectral power in the gamma band^65^. The procedure for an unbiased detection of upstates is showed in Supplementary Fig. 1. In brief, we computed the short-time Fourier transform (STFT) in the 40–90 Hz frequency band with an overlapped Blackman window of 0.2 s. Spectrograms were normalized to the mean FFT in order to make different frequencies comparable in power^65^. Gamma-band activity (GBA) was estimated as the sum of the normalized STFT components in the considered frequency range. GBA was smoothed with a sliding window of 80 ms and logarithmically scaled. The time histogram of log(GBA) was bimodal, reflecting the distribution of gamma band-activity during up and down states. The threshold for the discrimination of up states was set to 50% of the peak-to-peak interval. A cut-off in the minimum up (down) states duration was set to 100 ms, and up (down) states shorter than the cut-off were assigned to the ongoing down (up) state (Supp. Fig. 1d).

To detect ISs the LFP signal a threshold was fixed at 3SD from mean. Peri-IS spectrograms were calculated using MATLAB STFT function with an overlapping Blackman window of 200 ms. Spectrograms were then normalized by the average LFP spectrum far (>2 sec) from IS events.

The track was high-pass filtering (200 Hz) for the spike detection on loose-patch recordings; a threshold of 3 SDs was used to detect spike events. Cell recordings that included less than 100 interictal spikes were excluded from subsequent analysis.

VEP amplitudes were calculated as the mean voltage in a window of 400 ms starting from 100 ms after stimulus presentation. The temporal distribution of amplitude values was smoothed with a Gaussian kernel of 100 ms to generate the temporal evolution of VEP amplitudes reported in Fig. 5d; two animals were excluded because of the low number of events.

## Additional Information

**How to cite this article**: Petrucco, L. *et al*. Epileptiform activity in the mouse visual cortex interferes with cortical processing in connected areas. *Sci. Rep.* **7**, 40054; doi: 10.1038/srep40054 (2017).

**Publisher's note:** Springer Nature remains neutral with regard to jurisdictional claims in published maps and institutional affiliations.

## Supplementary Material

supplementary Figure 1
