# Corrigendum: Epileptiform activity in the mouse visual cortex interferes with cortical processing in connected areas

**DOI:** 10.1038/srep43808

**Published:** 2017-03-14

**Authors:** L. Petrucco, E. Pracucci, M. Brondi, G. M. Ratto, S. Landi

Scientific Reports 7: Article number: 4005410.1038/srep40054; published online: 01 10 2017; updated: 03 14 2017

This Article contains typographical errors in the Results section under subheading ‘ISs fragment the up states in the opposite hemisphere’.

“The presence of ISs in Hem 2 was associated with a change of the statistics of the slow-wave oscillations in Hem 1; in particular, as shown in Fig. 3c,d, the frequency of up states increased from 0.70 ± 0.06 Hz to 0.81 ± 0.06 Hz (paired t-test, n = 8, p = 0.007), while their duration decreased from 0.74 ± 0.04 s to 0.64 ± 0.03 s (paired t-test, n = 8, p = 0.003)”.

should read:

“The presence of ISs in Hem 2 was associated with a change of the statistics of the slow-wave oscillations in Hem 1; in particular, as shown in Fig. 3c,d, the frequency of up states increased from 0.57 ± 0.03 Hz to 0.64 ± 0.02 Hz (paired t-test, n = 8, p = 0.004), while their duration decreased from 0.74 ± 0.04 s to 0.64 ± 0.03 s (paired t-test, n = 8, p = 0.003)”.

