# Supplementary figures and images for: Epileptiform activity in the mouse visual cortex interferes with cortical processing in connected areas
Source: Sci Rep. 2017 Jan 10;7:40054. doi: 10.1038/srep40054 (PMC5223162; doi:10.1038/srep40054)

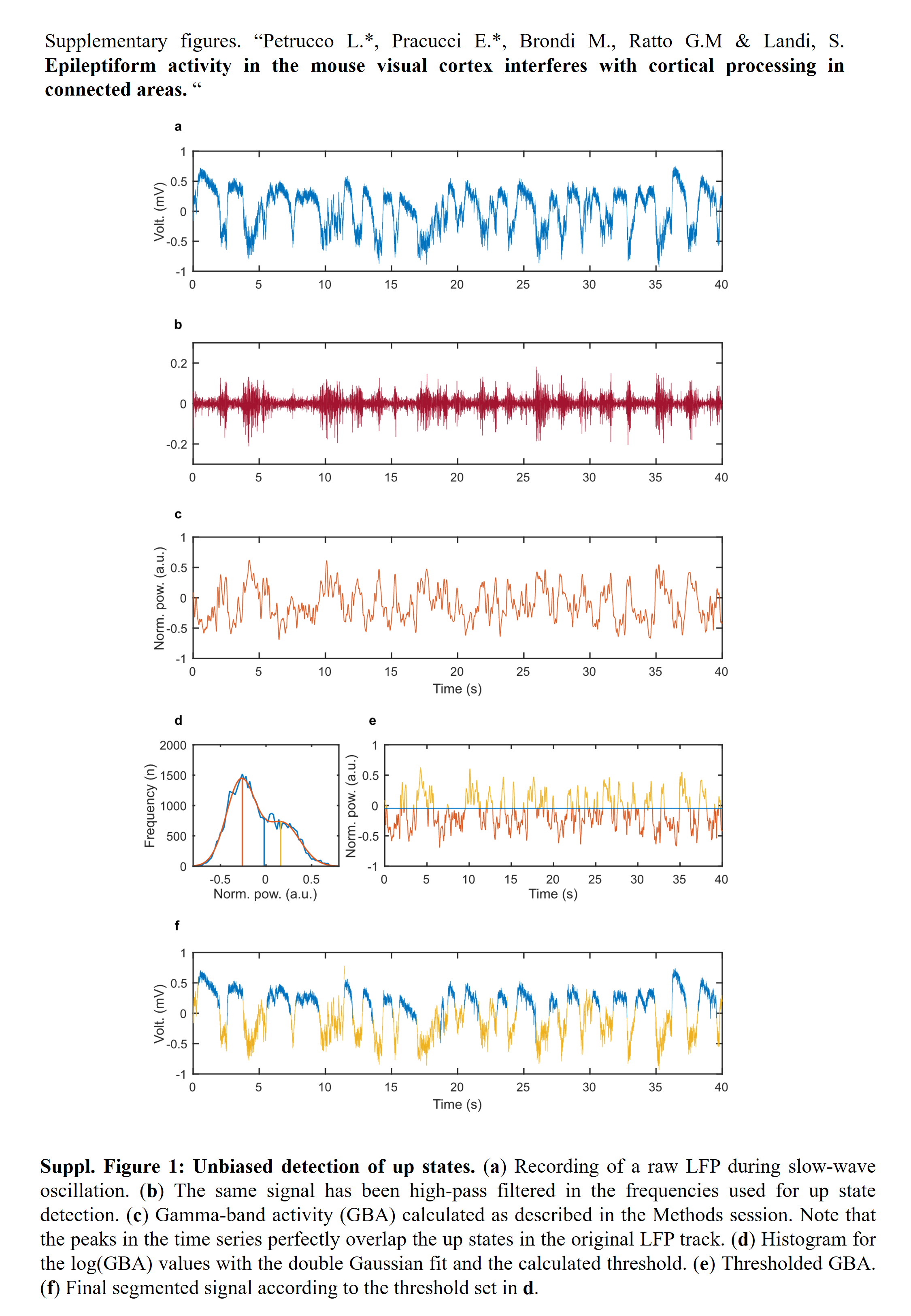

Supplement: supplementary Figure 1 [file srep40054-s1.tiff]
